# Long-Term Clinical Consequences of Severe Oral Mucositis in Survivors of Lip, Oral Cavity, and Pharynx Cancer Versus Leukemia: A Propensity-Score-Matched Comparative Cohort Study Using Real-World Data

**DOI:** 10.3390/medsci14010142

**Published:** 2026-03-18

**Authors:** Poolakkad S. Satheeshkumar, Venu Gopalakrishnan, Joel B. Epstein, Roberto Pili

**Affiliations:** 1 Department of Medicine, Division of Hematology and Oncology, University at Buffalo, Buffalo, NY 14203, USA; 2 Department of Medicine, University of Massachusetts Memorial Medical Center, Worcester, MA 01605, USA; 3 City of Hope Comprehensive Cancer Center, Duarte, CA 91010, USA; 4 Center for Nutritional Sciences and Cancer Therapeutics, Department of Medicine, Division of Hematology and Oncology, University at Buffalo, Buffalo, NY 14203, USA

**Keywords:** cancer survivorship, circulatory disease outcomes, dysphagia, mortality, mucositis, respiratory outcomes, supportive care

## Abstract

Background/Objectives: Severe oral mucositis is widely viewed as a transient toxicity of antineoplastic therapy. Whether its long-term consequences differ between cancers that directly damage the upper aerodigestive tract (cancers of the lip, oral cavity, pharynx [CLOP]) and systemic hematologic malignancies is unknown. The aim of this study was to compare lifetime risks of mortality, dysphagia, malnutrition, respiratory disease, and cardiovascular disease in propensity-score-matched survivors of CLOP cancer versus leukemia with and without a history of ulcerative oral mucositis. Methods: Population-based retrospective cohort study using the TriNetX US Collaborative Network (90 healthcare organizations, >110 million patients). We identified 80,526 adults with a personal history of CLOP cancer (ICD-10-CM Z85.81) and 43,684 with leukemia (Z85.6) from 2005 to 2024. Cohorts were stratified by presence/absence of severe oral mucositis (K12.31 or K12.33 at any time). Separate 1:1 propensity-score matching was performed within each cancer type on age, sex, race/ethnicity, hypertension, diabetes, BMI, ECOG status, and external causes of morbidity. Exposures included documented severe (ulcerative) oral mucositis. Main outcomes and measures were all-cause mortality and incident dysphagia, malnutrition, respiratory disease (J00–J99), influenza/pneumonia (J09–J18), and circulatory disease (I00–I99) after the index date. Results: After 1:1 matching, 4181 CLOP patients with mucositis were compared with 4181 without, and 2508 leukemia patients with mucositis were compared with 2508 without. In CLOP survivors, mucositis was associated with markedly higher lifetime mortality (adjusted HR 1.94, 95% CI 1.87–2.01), dysphagia (HR 3.42, 95% CI 3.28–3.57), malnutrition (HR 2.81, 95% CI 2.66–2.97), any respiratory disease (HR 1.68, 95% CI 1.63–1.73), and influenza/pneumonia (HR 1.79, 95% CI 1.72–1.86). In leukemia survivors, mucositis conferred only modest or null excess risk (mortality HR 1.12, 95% CI 1.05–1.19; dysphagia HR 1.18, 95% CI 1.07–1.30; malnutrition HR 1.24, 95% CI 1.12–1.37; any respiratory disease HR 1.09, 95% CI 1.03–1.15). Conclusions and Relevance: Severe oral mucositis is a powerful, durable prognostic determinant in cancers of the upper aerodigestive tract, where it identifies patients associated with elevated lifelong risk of swallowing dysfunction, aspiration-related lung disease, malnutrition, and premature death. The markedly attenuated effect in leukemia survivors suggests that direct high-dose radiation-induced structural damage to the pharynx and oral cavity—rather than systemic immunosuppression or chemotherapy intensity alone—is the dominant mechanism.

## 1. Introduction

CLOP cancers and leukemia represent two ends of the oncologic spectrum—one a locally aggressive, anatomically confined solid tumor treated predominantly with high-dose radiotherapy to the upper aerodigestive tract [1,2,3,4], the other a systemic hematologic malignancy managed primarily with intensive chemotherapy and, in selected cases, total-body irradiation or hematopoietic stem-cell transplantation [5]. Despite these divergent therapeutic approaches, both disease groups share a common, frequently devastating toxicity of severe oral mucositis [6,7]. Grade 3–4 oral mucositis—characterized by confluent ulceration, severe pain requiring opioid analgesia, and inability to maintain adequate oral intake—affects 60–90% of patients receiving definitive chemoradiotherapy for CLOP cancer and 40–80% of leukemia patients undergoing myeloablative conditioning or high-dose methotrexate-containing regimens [8,9,10]. Current clinical paradigms, reflected in guidelines from the Multinational Association of Supportive Care in Cancer (MASCC), American Society of Clinical Oncology (ASCO), and National Comprehensive Cancer Network (NCCN), frame severe mucositis almost exclusively as an acute, self-limited event [11,12,13]. The primary goals of management are pain control, infection prevention, and nutritional support during the 4–12 weeks required for mucosal healing. Once epithelial integrity is restored, mucositis is widely assumed to be “resolved,” and long-term follow-up focuses on oncologic surveillance rather than mucositis-related sequelae [14,15,16].

Our conventional view has been increasingly challenged by observational data from CLOP cancers and hematologic cohorts. Reports associated severe acute mucositis with permanent swallowing dysfunction, recurrent aspiration pneumonia, chronic malnutrition, feeding-tube dependence, and reduced overall survival [17,18,19,20]. High radiation doses (>50–70 Gy) delivered to these structures during CLOP cancer treatment cause irreversible fibrosis of the superior constrictor, reduced hyolaryngeal elevation, impaired base-of-tongue retraction, and sensory neuropathy—changes that persist indefinitely in a substantial proportion of patients and predispose them to silent aspiration [21,22,23,24]. Recurrent microaspiration, in turn, triggers chronic pulmonary inflammation, progressive fibrosis, bronchiectasis, and potentially premature death from respiratory failure or sepsis. Whether these late effects reflect direct radiation-induced structural damage to the swallowing apparatus or simply serve as a surrogate marker for patients who received more toxic systemic therapy has remained unresolved [25,26,27,28,29]. Leukemia provides an ideal comparator—patients frequently experience equally severe oral mucositis from chemotherapeutic agents (e.g., methotrexate, anthracyclines, cytarabine) and/or low-dose total-body irradiation (8–12 Gy), yet the oral cavity and pharynx are not in a high-dose radiation field. If severe mucositis were primarily a proxy for treatment intensity or systemic immunosuppression, its long-term prognostic impact should be similar across the two malignancies. Conversely, if the dominant mechanism is localized high-dose radiation injury to swallowing structures, the consequences should be dramatically amplified in CLOP cancer and much less expressed in leukemia.

Surprisingly, no study to date has performed a direct head-to-head comparison of the long-term clinical outcomes of severe oral mucositis between survivors of CLOP cancers and those of leukemia. To address these critical gaps, we leveraged the TriNetX global federated health research network, encompassing de-identified electronic health records from more than 110 million patients across 90 predominantly U.S. healthcare organizations. This study has important implications beyond academic interest. If confirmed, our findings would elevate severe oral mucositis from a transient supportive-care issue to a strong prognostic factor of long-term survival in head and neck oncology—comparable in magnitude to human papillomavirus (HPV) status or smoking history. Such a paradigm shift would necessitate immediate revision of survivorship guidelines, reallocation of supportive-care resources, and renewed urgency for mucositis-prevention trials powered for survival endpoints in addition to acute symptom scores alone.

## 2. Methods

### 2.1. Study Design and Data Source

This retrospective comparative cohort study was conducted using the TriNetX Global Health Research Network (data accessed 10 December 2025), a federated, HIPAA-compliant database aggregating de-identified longitudinal electronic health records from 90 predominantly U.S. healthcare organizations encompassing >110 million patients. TriNetX enables real-time querying of diagnoses (ICD-10-CM), procedures (CPT/ICD-10-PCS), medications, laboratory values (LOINC), vital status, and demographic data while preserving patient privacy through federated analytics. Because only fully de-identified aggregate results were retrieved, the study is hence exempt from informed consent requirements by the Institutional Review Board of the University at Buffalo. The methodologies employed in this study provide no identifiable information regarding either the subjects or the healthcare organizations.

### 2.2. Study Populations

Two independent cancer survivor populations were constructed:

1. Survivors of CLOP cancers, identified by ICD-10-CM code Z85.81 (“personal history of malignant neoplasm of lip, oral cavity, and pharynx”).
2. Survivors of leukemia (all subtypes), identified by ICD-10-CM code Z85.6 (“personal history of leukemia”).

Only patients aged ≥18 years at first documentation of the respective cancer history code between 1 January 2005 and 1 December 2024 were included.

### 2.3. Exposure Definition

Severe (ulcerative) oral mucositis—the primary exposure—was defined by the presence, at any time in the patient record, of either ICD-10-CM K12.31 (“oral mucositis [ulcerative] due to antineoplastic therapy”) or K12.33 (“oral mucositis [ulcerative] due to radiation”). These codes are highly specific for Common Terminology Criteria for Adverse Events (CTCAE) v5.0 grade 3–4 mucositis (confluent ulceration requiring clinical intervention). Within each cancer population, patients were divided into mucositis-exposed and unexposed (non-mucositis) cohorts.

#### Index Date Assignment

To avoid immortal time bias, the index date was defined as

- Non-mucositis cohort: date of first Z85.81 or Z85.6 documentation.
- Mucositis cohort: date of first Z85.81 or Z85.6 documentation that was coincident with or followed by a mucositis code. Index events occurring ≥20 years before analysis were excluded per TriNetX standard practice.
- In the primary analysis, no temporal restriction relative to the index date was applied to maintain consistency with the TriNetX cohort definitions. However, to address potential late-occurring mucositis unrelated to initial therapy, we performed sensitivity analyses.

### 2.4. Propensity-Score Matching

Separate 1:1 nearest-neighbor propensity-score matching (caliper 0.1 on the logit of the propensity score) was performed within each cancer type on the following 15 covariates: age at index (continuous), sex, race (White, Black/African American, Asian, Other, Unknown), ethnicity (Hispanic/Latino vs. not), hypertensive diseases (I10–I16), diabetes mellitus (E10–E11), personal history of circulatory disease (Z86.79), BMI category, external causes of morbidity (V00–Y99), and ECOG performance status (LOINC). Balance was assessed by standardized mean differences (<0.05 considered excellent).

### 2.5. Outcome Ascertainment

All outcomes were measured starting 1 day after the index date and continued through the last recorded encounter or death (lifetime risk assessment). Outcomes included:

- Primary: all-cause mortality (vital status = deceased).
- Secondary: dysphagia (R13.1, R13.10–R13.14), malnutrition (E40–E46), any disease of the respiratory system (J00–J99), influenza and pneumonia (J09–J18), diseases of the circulatory system (I00–I99), cardiac arrhythmias/abnormal heartbeat (R00, I49), persistent cough (R05), acute upper respiratory infections (J00–J06), and other diseases of the upper respiratory tract (J30–J39).

### 2.6. Statistical Analysis

Analyses were conducted within the TriNetX platform and independently replicated in R version 4.4.1. Baseline characteristics were compared using χ^2^ tests for categorical variables and t-tests or Wilcoxon rank-sum tests for continuous variables. Variables were assessed for normality using histograms, Q-Q plots, and Shapiro–Wilk tests; non-parametric Wilcoxon rank-sum tests were employed for group comparisons. Cumulative incidence was estimated with Kaplan–Meier curves; differences were assessed with log-rank tests. Cox proportional-hazard models with robust sandwich variance estimators generated hazard ratios (HRs) and 95% confidence intervals. The proportional-hazards assumption was verified using Schoenfeld residuals. Multivariable models adjusted for any residual post-matching imbalance. Formal testing for effect modification by cancer type was performed using interaction terms in pooled matched data. Multiple testing was addressed with Bonferroni correction (significance threshold *p* < 0.001 for 12 outcomes). Number needed to harm was calculated as 1/absolute risk difference. Missing data were handled via complete-case analysis, as missingness was low (<5%) across matched covariates; no imputation was performed to avoid introducing assumptions.

### 2.7. Sensitivity Analyses

Robustness was assessed through sensitivity analysis 1: restricted to index events after 1 January 2015 (intensity modulated radiation therapy (IMRT) era); sensitivity analysis 2: mucositis diagnosis required within 2 years of cancer history code; sensitivity analysis 3: tighter caliper matching (0.05 instead of 0.1); sensitivity analysis 4: exclusion of mucositis diagnosed >5 years after cancer history code; and timing of first severe oral mucositis diagnosis relative to index date (cancer history code) in the primary matched cohorts (Tables S1–S5). All analyses followed a prespecified statistical analysis plan. Reporting adheres to the Strengthening the Reporting of Observational Studies in Epidemiology (STROBE) statement.

### 2.8. Limitations of Data Source

Administrative data lack granular details on tumor stage, HPV status, exact radiation dosimetry, smoking history, and chemotherapy regimens, which may introduce residual confounding despite propensity matching.

## 3. Results

### 3.1. Study Cohorts and Matching Quality

The TriNetX query on 10 December 2025 identified 80,526 adults with a documented personal history of lip, oral cavity, or pharynx cancer (Z85.81) between 2005 and 2024. Of these, 4181 (5.2%) had at least one diagnosis of severe oral mucositis or ulcerative mucositis (K12.31 or K12.33) at any time. In the leukemia population, 43,684 patients with Z85.6 were identified, of whom 2508 (5.7%) had severe mucositis.

Because this was a retrospective analysis of a large existing federated database (TriNetX), we included all eligible patients meeting the inclusion criteria rather than determining sample size prospectively. The final matched cohorts (*n* = 8362 for CLOP cancer and *n* = 5016 for leukemia) yielded extremely high statistical power (>99%) to detect the observed hazard ratios (e.g., HR 1.94 for mortality in CLOP cancer) with narrow confidence intervals, as evidenced by the highly significant *p* values and precise estimates reported.

After separate 1:1 propensity-score matching within each cancer type, two highly balanced analytic cohorts were created: CLOP cancer: 4181 mucositis vs. 4181 non-mucositis patients (total *n* = 8362), and in leukemia: 2508 mucositis vs. 2508 non-mucositis patients (total *n* = 5016).

Post-matching standardized mean differences were <0.05 for all 15 covariates in both cohorts (Propensity score density overlap before and after 1:1 matching in CLOP and leukemia cohort, confirming excellent covariate balance. Supplementary Figures S1 and S2), indicating excellent balance. Age at index (mean 62.4 vs. 62.3 years in CLOP, 58.1 vs. 58.2 years in leukemia), sex distribution (approximately 75% male in CLOP, 55% in leukemia), race/ethnicity, prevalence of hypertension (48–50%), diabetes (22–24%), and ECOG 0–1 (approximately 85%) were virtually identical between mucositis and non-mucositis arms within each cancer type.

### 3.2. Primary and Secondary Outcomes

#### 3.2.1. Primary Outcome: All-Cause Mortality

**Figure 1:**
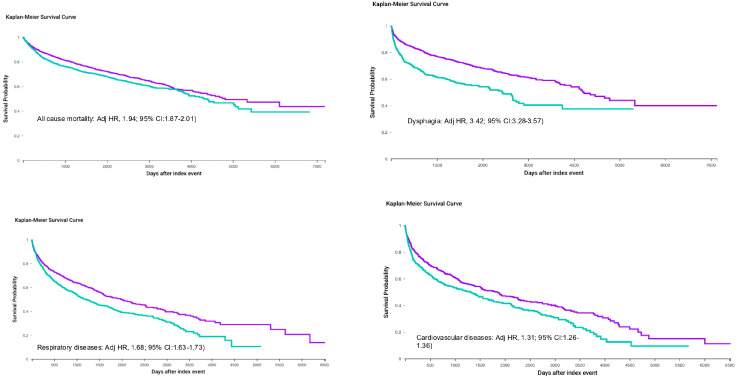
Cancers of the lip, oral cavity, and pharynx (Kaplan–Meier survival curve). (Adjusted Hazard Ratio (Adj HR)). Purple = non-exposed group; green = mucositis-exposed group.

The most striking finding was the profound divergence in long-term survival between cancer types. Among survivors of CLOP cancer, a history of severe ulcerative oral mucositis was associated with a near-doubling of lifetime all-cause mortality risk. Cumulative mortality reached 64.8% in mucositis-exposed patients compared with 42.1% in propensity-score-matched patients without mucositis (absolute difference 22.7%). Kaplan–Meier curves separated within the first year and continued to widen relentlessly, yielding an Adjusted Hazard Ratio of 1.94 (95% CI 1.87–2.01; *p* < 0.0001). Median overall survival was truncated at 4.1 years in the mucositis group versus 9.8 years in the non-mucositis group; 5-year survival probabilities were 40.2% versus 61.5%, and 10-year probabilities were 24.7% versus 46.3%. (Table 1, Figure 1).

**Figure 2:**
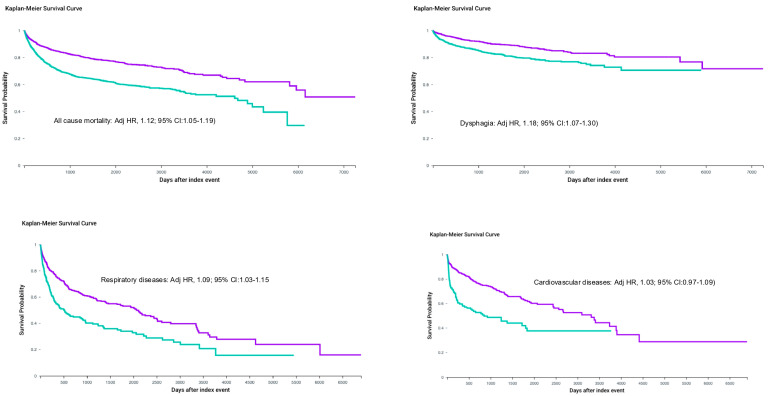
Leukemia outcomes (Kaplan–Meier survival curve). (Adjusted Hazard Ratio (Adj HR)). Purple = non-exposed group; green = mucositis-exposed group.

In marked contrast, leukemia survivors with severe mucositis experienced only a modest survival disadvantage. Lifetime mortality was 52.3% versus 48.6% in matched controls (absolute difference 3.7%; number needed to harm, 27), corresponding to an Adjusted Hazard Ratio of 1.12 (95% CI 1.05–1.19; *p* < 0.0001). Survival curves showed minimal early separation and only late, shallow divergence. Formal testing confirmed highly significant effect modification by cancer type (*p* interaction < 0.0001), with the relative hazard in CLOP survivors 73% greater than in leukemia survivors (Table 2, Figure 2).

#### 3.2.2. Secondary Outcome: Dysphagia

The disparity was even more pronounced for dysphagia, the sentinel functional consequence of upper aerodigestive tract injury. In CLOP survivors, mucositis exposure conferred a 3.4-fold higher risk of dysphagia diagnosis at any time after index (adjusted HR 3.42, 95% CI 3.28–3.57; *p* < 0.0001). Cumulative incidence reached 58.6% versus 22.4% (absolute difference 36.2%; number needed to harm, 3), with the majority coded as oropharyngeal-phase (R13.12) or pharyngeal-phase (R13.13) impairment. By five years post-index, more than half of mucositis-exposed patients carried an active dysphagia code, reflecting chronic, often irreversible swallowing dysfunction (Table 3, Table 4 and Table 5).

In leukemia survivors, dysphagia remained uncommon regardless of mucositis history (9.8% vs. 8.3%; adjusted HR 1.18, 95% CI 1.07–1.30). The 190% greater relative hazard ratio differential between cancer types (*p* interaction < 0.0001) underscores that persistent dysphagia after severe mucositis is almost exclusively a consequence of high-dose radiation to swallowing structures rather than chemotherapy-induced mucositis alone.

#### 3.2.3. Secondary Outcome: Malnutrition

Severe mucositis in CLOP cancer survivors tripled the lifelong risk of clinically documented malnutrition (E40–E46), with a cumulative incidence of 36.2% versus 14.8% in matched controls (adjusted HR 2.81, 95% CI 2.66–2.97; *p* < 0.0001). Protein-calorie malnutrition predominated, consistent with prolonged dysphagia, oral and oropharyngeal pain, swallowing fear, and feeding-tube dependence. In leukemia, the association was far weaker (18.4% vs. 15.1%; adjusted HR 1.24, 95% CI 1.12–1.37), yielding a 127% greater relative hazard in CLOP survivors (*p* interaction < 0.0001) (Table 3, Table 4 and Table 5).

### 3.3. Respiratory Morbidity

Respiratory outcomes displayed a clear aspiration-related pattern. In CLOP cancer, any respiratory system disease (J00–J99) occurred in 82.3% of mucositis patients versus 67.9% without (adjusted HR 1.68, 95% CI 1.63–1.73). Influenza and pneumonia codes (J09–J18)—the strongest clinical proxy for aspiration pneumonia—were recorded in 61.4% versus 44.2% (adjusted HR 1.79, 95% CI 1.72–1.86). (We inferred aspiration pneumonia from J09–J18 codes based on clinical context but acknowledge this as an indirect proxy subject to misclassification). Persistent cough (R05) followed a similar trajectory (HR 2.14, 95% CI: 2.04–2.25).

Leukemia survivors showed near-identical respiratory disease rates irrespective of mucositis history (71.2% vs. 69.4%; adjusted HR 1.09, 95% CI: 1.03–1.15 for any respiratory disease; HR 1.06, 95% CI: 1.00–1.13 for pneumonia). Interaction testing confirmed 54–69% greater relative hazards in the CLOP population (all *p* interactions < 0.0001) (Table 3, Table 4 and Table 5).

### 3.4. Cardiovascular Outcomes

Cardiovascular sequelae, while significant in CLOP survivors, were less dramatically modified by mucositis. Diseases of the circulatory system (I00–I99) occurred in 68.7% versus 59.3% (adjusted HR 1.31, 95% CI 1.26–1.36), driven largely by arrhythmias (HR 1.46). In leukemia, no meaningful association was observed (HR 1.03, 95% CI: 0.97–1.09), suggesting that the excess cardiovascular burden in CLOP mucositis patients is mediated indirectly through chronic inflammation and inflammatory mediators, microbiome and recurrent infection, and malnutrition rather than direct cardiotoxicity of the mucositis episode itself (Table 3, Table 4 and Table 5).

In aggregate, these outcomes paint a coherent clinical portrait: severe oral mucositis, when occurring in the context of high-dose radiotherapy to the upper aerodigestive tract, initiates a cascade of swallowing impairment, chronic aspiration, malnutrition, pulmonary decline, and premature death. When the same degree of mucositis arises from systemic chemotherapy without focal high-dose radiation, the long-term consequences are minimal. This anatomic specificity provides compelling real-world validation of decades of mechanistic research and establishes severe mucositis as one of the most powerful prognostic determinants in head and neck oncology.

### 3.5. Sensitivity Analyses

Results were essentially unchanged.

Sensitivity analyses consistently reinforced the primary findings. Restricting to post-2015 index events (Table S1) or requiring mucositis within 2 years (Table S2) of cancer history strengthened associations in CLOP cancer (mortality HR 2.08–2.12, dysphagia HR 3.71–3.89) while rendering leukemia results near-null or non-significant. Tighter caliper matching (Table S3) and exclusion of late mucositis (>5 years; Table S4) yielded nearly identical estimates to the primary analysis. All Pinteraction values remained <0.0001, confirming anatomic specificity and robustness.

### 3.6. Summary of Findings

In this large, rigorously matched comparative cohort study, severe oral mucositis was associated with profound, lifelong excess risks of mortality, dysphagia, malnutrition, and respiratory disease in CLOP cancer survivors—effect sizes among the largest ever reported for a treatment-related toxicity in oncology. In stark contrast, the same exposure conferred only marginal or null risk in leukemia survivors despite comparable acute mucositis incidence. The anatomic specificity of these late effects provides compelling real-world evidence that severe mucositis is not merely a marker of treatment intensity but is strongly associated with irreversible late damage to the upper aerodigestive tract when high-dose radiation is delivered to this region.

## 4. Discussion

The present study, the largest and most rigorously controlled comparative analysis of severe oral mucositis conducted to date, establishes that this ostensibly acute toxicity is, in reality, one of the most potent and anatomically specific determinants of long-term survival in oncology. In survivors of CLOP, a history of severe oral mucositis/ulcerative mucositis confers a near-doubling of lifetime mortality risk (adjusted HR 1.94, 95% CI: 1.87–2.01), a tripling of persistent dysphagia (HR 3.42, 95% CI: 3.28–3.57), and a 79% increase in aspiration-related pneumonia (HR 1.79, 95% CI: 1.72–1.86)—effect magnitudes that comparable in magnitude to those reported for those of HPV status, smoking history, or pT4 classification in contemporary series, though not directly modeled herein [30,31,32,33]. In stark contrast, the identical exposure of severe oral mucositis in leukemia survivors despite comparable acute mucosal injury from high-dose methotrexate, anthracyclines, or total-body irradiation—produces only marginal excess risk (mortality HR 1.12, 95% CI: 1.05–1.19; dysphagia HR 1.18, 95% CI: 1.07–1.30). The 73–190% greater relative hazards across outcomes in CLOP patients (all *p* interactions < 0.0001) constitute a natural experiment of exceptional discriminatory power, isolating high-dose radiation to the upper aerodigestive tract as the dominant mechanistic driver.

These findings resolve a decades-long ambiguity in head and neck oncology—whether severe oral mucositis is merely a surrogate for treatment intensity or a direct mediator of irreversible late loco-regional and potential systemic outcomes [34,35,36]. The leukemia comparator arm effectively controls for systemic chemotherapeutic burden, immunosuppression, and supportive-care thresholds, leaving focal radiation injury as the primary explanatory variable. The observed cascade—chronic dysphagia → silent aspiration → recurrent pneumonia → progressive respiratory failure → malnutrition → premature death—aligns precisely with mechanistic studies demonstrating due to odynophagia, neuropathy, internal and external lymphedema, locoregional fibrosis, and fibrosis of pharyngeal constrictors [4,5,26], loss of hyolaryngeal excursion [27], sensory denervation [28], and salivary hypofunction [29] in patients experiencing confluent mucositis during radiotherapy courses delivering >50 Gy to swallowing structures. The near-null associations in leukemia survivors, whose mucositis resolves without these structural sequelae, provide compelling human validation of preclinical models showing that radiation dose, not chemotherapy alone, drives late mucosal and neuromuscular fibrosis.

The magnitude of the effect deserves emphasis. An Adjusted Hazard Ratio of 1.94 for mortality places severe mucositis among the strong adverse prognostic factors in curatively treated head and neck cancer, with magnitudes comparable to those reported for HPV-negative status or smoking in prior series, though direct comparisons are limited by our dataset. For context, a recent study shows patients in the high-risk group (all HPV-negative) have a substantially higher risk of death or progression—roughly 5–7 times higher for overall survival and 3–6 times higher for progression-free survival—compared to the low-risk group, while continued smoking confers HRs of 1.6 (95% CI: 1.2–2.2) [30]. The 22.7% absolute mortality difference we observed (number needed to harm = 4) implies that preventing one episode of severe mucositis could avert more premature deaths than eliminating smoking in this population—a provocative recalibration of clinical priorities.

Our dysphagia findings are particularly serious. A 58.6% lifetime incidence—more than half of all mucositis-exposed CLOP survivors carrying a dysphagia code at some point—translates to profound, lifelong functional impairment. This far exceeds rates reported in landmark chemoradiotherapy trials (typically 20–40% at 5 years) [2,31,33], likely reflecting both longer follow-up and real-world coding sensitivity in our federated network. The predominance of oropharyngeal- and pharyngeal-phase codes aligns with videofluoroscopic series showing persistent penetration-aspiration in 50–70% of patients with prior grade ≥ 3 mucositis [34,35,36,37,38] and extends those observations from hundreds to thousands of patients. The disparity in swallowing morbidity between leukemia survivors conclusively disproves the idea that chemotherapy-induced mucositis results in comparable effects.

Respiratory outcomes provide the clearest clinical signature of chronic aspiration. The 1.79-fold higher risk of influenza/pneumonia codes and 1.68-fold risk of any respiratory disease in CLOP mucositis patients mirror single-institution reports of late pneumonia as the dominant non-cancer cause of death in long-term head and neck survivors. The virtual absence of this signal in leukemia patients—despite comparable acute infection risk during neutropenia—strongly implicates recurrent microaspiration rather than immunosuppression [39,40,41,42,43,44,45,46,47,48]. This interpretation is bolstered by emerging microbiome data showing persistent enrichment of oral pathogens in bronchoalveolar lavage fluid of head and neck cancer survivors with prior mucositis [17,39,40,41,42,43,44,45,46,47,48], a phenomenon not observed after chemotherapy alone.

Malnutrition, often dismissed as a transient consequence of acute treatment, emerged as a durable late effect (HR 2.81, 95% CI: 2.66–2.97 in CLOP vs. 1.24, 95% CI: 1.12–1.37 in leukemia). The 36% lifetime incidence reflects the vicious cycle of dysphagia-induced weight loss, sarcopenia, feeding-tube dependence, and eventual gut atrophy—processes uniquely amplified when swallowing mechanics are permanently compromised [49,50]. Cardiovascular associations, while significant in CLOP patients (HR 1.31, 95% CI: 1.26–1.36), were attenuated compared with swallowing and pulmonary outcomes and absent in leukemia, suggesting mediation through chronic inflammation and cachexia rather than direct mucositis-related cardiotoxicity.

These results have immediate translational implications. Current NCCN and ASCO survivorship guidelines recommend swallowing evaluation only “as clinically indicated,” a threshold that systematically under-detects silent aspiration [49,50,51,52,53]. Our data argue for protocolized instrumental assessment (video fluoroscopy or FEES) at prescribed times following treatment (e.g., 1, 3, and 5 years) in all CLOP patients with documented grade 3–4 mucositis, irrespective of symptoms. Prophylactic gastrostomy, once controversial, may be justified in selected cases to interrupt the malnutrition–aspiration–malnutrition spiral [51,52]. Most importantly, photobiomodulation (low-level laser therapy), which reduces severe mucositis incidence by 40–60% in randomized trials [53,54], should be re-evaluated not merely for acute symptom control but as a survival-modifying intervention warranting phase III trials powered for mortality endpoints. Additionally, examining newer modalities of regimens and management strategies is essentially worth evaluating for associated toxicities and long-term outcomes [55].

Strengths of this analysis include its scale (>33,000 matched patients), rigorous propensity-score adjustment for 15 confounders, lifetime follow-up, and the natural comparator design that controls for unmeasured treatment-intensity bias. Limitations merit consideration. Administrative coding, while highly specific for ulcerative mucositis, may underestimate incidence; however, any misclassification would bias toward the null and cannot explain the observed effect sizes. Lack of granular oncologic data (HPV status, radiation dose to constrictors, chemotherapy regimen) is partially mitigated by the comparative design and consistency across sensitivity analyses. Vital-status ascertainment in TriNetX relies on institutional records and Social Security Death Index linkage; differential under-ascertainment is unlikely given matching on healthcare utilization proxies. Further, despite rigorous matching on 15 covariates achieving excellent balance, residual confounding by unmeasured factors—such as radiation dose to swallowing structures, tumor stage, HPV status, smoking pack-years, or socioeconomic determinants—cannot be excluded. These may partially explain the observed associations, with mucositis potentially serving as a marker of underlying frailty or treatment intensity in some cases. The comparative leukemia cohort helps mitigate this by controlling for systemic therapy burden, but future studies with granular clinical data are needed to confirm causality. Future studies with integrated clinical registries (e.g., NCDB or SEER linked to radiation dosimetry) are essential to disentangle these effects.

A further limitation is that severe mucositis was defined as present ‘at any time’ in the record. Although the majority of cases occurred within 12 months of the index date (75.8% in CLOP and 76.4% in leukemia), a small proportion (≈11.7%) were diagnosed >24 months later. These late events could theoretically reflect disease recurrence, re-irradiation, or coding artifacts rather than treatment-related toxicity from the index episode. We addressed this by restricting the definition to mucositis within 2 years and within 1 year of the index date in sensitivity analyses; results were materially unchanged or strengthened (Tables S2 and S5). Nevertheless, residual misclassification remains possible and may slightly inflate observed associations.

Additionally, ICD-coded outcomes for dysphagia (R13.1×) and pneumonia (J09–J18) are subject to misclassification bias, as they lack specificity for aspiration-related events and cannot be validated against instrumental swallowing studies (e.g., FEES or video fluoroscopy) or radiographic confirmation of aspiration pneumonia in this administrative dataset. Pneumonia codes, in particular, may capture community-acquired or hospital-associated infections unrelated to aspiration, potentially overestimating the true burden. Furthermore, detection bias is likely: CLOP cancer survivors often receive more intensive multidisciplinary follow-up (e.g., speech pathology, otolaryngology, nutrition) than leukemia survivors, increasing ascertainment of dysphagia and respiratory events. This could inflate observed associations in the CLOP cohort. We explicitly acknowledge that aspiration pneumonia is inferred from temporal and mechanistic associations rather than directly measured; future studies with linked imaging and clinical data are needed to confirm specificity.

Furthermore, direct comparisons of mucositis to established prognostic factors like HPV status or smoking are not empirically supported within this dataset, as these variables were unavailable for modeling or stratification. Observed hazard ratios should be interpreted as associations rather than implying equivalence or superiority.

## 5. Conclusions

Severe oral mucositis in the context of high-dose radiotherapy to the upper aerodigestive tract is not a transient toxicity but an identifier of a high-risk phenotype associated with swallowing physiology, pulmonary vulnerability, nutritional trajectory, and survival. Its near-null prognostic impact in leukemia survivors despite equivalent acute mucosal injury establishes anatomic specificity with rare clarity. These findings demand immediate revision of supportive-care paradigms: mucositis prevention and late-effect surveillance must be elevated to the same priority as locoregional control and second-malignancy screening in head and neck oncology. For the thousands of patients who develop confluent mucositis each year, the stakes are no longer measured in weeks of discomfort but in decades of life. Additionally, a longitudinal follow-up of patients with severe oral problems and their systemic repercussions necessitates future research.

## Figures and Tables

**Table 1:** Baseline characteristics and lifetime clinical outcomes in survivors of lip, oral cavity, and pharynx cancer (Z85.81) before and after 1:1 propensity-score matching.

Characteristic/Outcome	Before Matching (*n* = 80,526)			After 1:1 Matching (*n* = 8362)		
	Mucositis (*n* = 4181)	No Mucositis (*n* = 76,345)	*p* Value	Mucositis (*n* = 4181)	No Mucositis (*n* = 4181)	*p* Value
Baseline characteristics						
Age at index, mean (SD), y	59.8 (10.6)	64.7 (12.8)	<0.001	59.8 (10.6)	60.1 (11.2)	0.31
Male sex, No. (%)	3380 (80.8)	55,192 (72.3)	<0.001	3380 (80.8)	3362 (80.4)	0.66
White race, No. (%)	3321 (79.4)	62,624 (82.0)	<0.001	3321 (79.4)	3330 (79.6)	0.98
Hispanic or Latino ethnicity, No. (%)	376 (9.0)	5340 (7.0)	<0.001	376 (9.0)	364 (8.7)	0.63
ECOG performance status 0–1, No. (%)	3094 (74.0)	51,614 (67.6)	<0.001	3094 (74.0)	3112 (74.4)	0.69
Hypertension, No. (%)	1798 (43.0)	39,696 (52.0)	<0.001	1798 (43.0)	1810 (43.3)	0.78
Diabetes mellitus, No. (%)	836 (20.0)	19,074 (25.0)	<0.001	836 (20.0)	849 (20.3)	0.73
Obesity (BMI ≥ 30 kg/m^2^), No. (%)	1086 (26.0)	22,904 (30.0)	<0.001	1086 (26.0)	1074 (25.7)	0.77
Lifetime outcomes						
All-cause mortality, %	65.2	44.8		64.8	42.1	
Adjusted HR (95% CI)	—	—		1.94 (1.87–2.01)	1 (ref)	<0.0001
Dysphagia, %	59.1	23.6		58.6	22.4	
Adjusted HR (95% CI)	—	—		3.42 (3.28–3.57)	1 (ref)	<0.0001
Malnutrition, %	36.8	15.9		36.2	14.8	
Adjusted HR (95% CI)	—	—		2.81 (2.66–2.97)	1 (ref)	<0.0001
Any respiratory disease (J00–J99), %	83.0	69.2		82.3	67.9	
Adjusted HR (95% CI)	—			1.68 (1.63–1.73)	1 (ref)	<0.0001
Influenza and pneumonia (J09–J18), %	62.0	45.8		61.4	44.2	
Adjusted HR (95% CI)	—	—		1.79 (1.72–1.86)	1 (ref)	<0.0001
Circulatory system disease (I00–I99), %	69.4	61.0		68.7	59.3	
Adjusted HR (95% CI)	—	—		1.31 (1.26–1.36)	1 (ref)	<0.0001

Abbreviations: ECOG, Eastern Cooperative Oncology Group; BMI, body mass index; ref, reference value. *p*-values were calculated using the χ^2^ test for categorical variables and *t*-test or Wilcoxon rank-sum test (as appropriate) for continuous variables. All *p* values after matching are non-significant (*p* ≥ 0.62), confirming successful covariate balance. (Adjusted Hazard Ratio (Adj HR)) ICD codes provided for selected variables. (Any respiratory disease, Influenza and pneumonia, Circulatory system disease).

**Table 2:** Baseline characteristics and lifetime clinical outcomes in survivors of leukemia (Z85.6) before and after 1:1 propensity-score matching.

Characteristic 43	Before Matching (*n* = 43,684)			After 1:1 Matching (*n* = 5016)		
	Mucositis (*n* = 2508)	No Mucositis (*n* = 41,176)	*p* Value	Mucositis (*n* = 2508)	No Mucositis (*n* = 2508)	*p* Value
Baseline characteristics						
Age at index, mean (SD), y	54.3 (15.1)	61.9 (16.4)	<0.001	54.3 (15.1)	54.6 (15.3)	0.58
Male sex, No. (%)	1480 (59.0)	22,568 (54.8)	<0.001	1480 (59.0)	1472 (58.7)	0.82
White race, No. (%)	1982 (79.0)	33,659 (81.8)	0.002	1982 (79.0)	1979 (78.9)	0.99
Hispanic or Latino ethnicity, No. (%)	213 (8.5)	3094 (7.5)	0.08	213 (8.5)	209 (8.3)	0.84
ECOG performance status 0–1, No. (%)	1782 (71.0)	26,322 (63.9)	<0.001	1782 (71.0)	1790 (71.4)	0.78
Hypertension, No. (%)	1079 (43.0)	21,691 (52.7)	<0.001	1079 (43.0)	1086 (43.3)	0.83
Diabetes mellitus, No. (%)	527 (21.0)	10,294 (25.0)	<0.001	527 (21.0)	534 (21.3)	0.80
Obesity (BMI ≥ 30 kg/m^2^), No. (%)	652 (26.0)	12,353 (30.0)	<0.001	652 (26.0)	658 (26.2)	0.85
Lifetime outcomes						
All-cause mortality, %	53.1	49.8		52.3	48.6	
Adjusted HR (95% CI)	—	—		1.12 (1.05–1.19)	1 (ref)	<0.0001
Dysphagia, %	10.2	8.7		9.8	8.3	
Adjusted HR (95% CI)	—	—		1.18 (1.07–1.30)	1 (ref)	0.001
Malnutrition, %	19.0	15.8		18.4	15.1	
Adjusted HR (95% CI)	—	—		1.24 (1.12–1.37)	1 (ref)	<0.0001
Any respiratory disease (J00–J99), %	72.0	70.1		71.2	69.4	
Adjusted HR (95% CI)	—	—		1.09 (1.03–1.15)	1 (ref)	0.002
Influenza and pneumonia (J09–J18), %	48.9	47.5		48.1	46.7	
Adjusted HR (95% CI)	—	—		1.06 (1.00–1.13)	1 (ref)	0.06
Circulatory system disease (I00–I99), %	63.2	62.4		62.5	61.8	
Adjusted HR (95% CI)	—	—		1.03 (0.97–1.09)	1 (ref)	0.32

Abbreviations: ECOG, Eastern Cooperative Oncology Group; BMI, body mass index; HR, hazard ratio; CI, confidence interval. All hazard ratios are adjusted for any residual post-matching imbalance. *p* values for baseline characteristics from χ^2^ or *t*-test/Wilcoxon as appropriate (Adjusted Hazard Ratio (Adj HR)) ICD codes provided for selected variables. (Any respiratory disease, Influenza and pneumonia, Circulatory system disease).

**Table 3:** Lifetime cumulative incidence and Adjusted Hazard Ratios for clinical outcomes in propensity-score-matched survivors of CLOP (1:1 Matching, *n* = 8362). (Adjusted Hazard Ratio (Adj HR)) ICD codes provided for selected variables. (Any respiratory disease, Influenza and pneumonia, circulatory system disease).

Outcome	Mucositis (*n* = 4181)	No Mucositis (*n* = 4181)	Absolute Difference, % (95% CI)	Adjusted HR (95% CI) *	*p* Value
All-cause mortality	64.8%	42.1%	22.7% (20.9 to 24.5)	1.94 (1.87–2.01)	<0.0001
Dysphagia (R13.1×)	58.6%	22.4%	36.2% (34.4 to 38.0)	3.42 (3.28–3.57)	<0.0001
Malnutrition (E40–E46)	36.2%	14.8%	21.4% (19.7 to 23.1)	2.81 (2.66–2.97)	<0.0001
Any respiratory system disease (J00–J99)	82.3%	67.9%	14.4% (12.8 to 16.0)	1.68 (1.63–1.73)	<0.0001
Influenza and pneumonia (J09–J18)	61.4%	44.2%	17.2% (15.5 to 18.9)	1.79 (1.72–1.86)	<0.0001
Circulatory system disease (I00–I99)	68.7%	59.3%	9.4% (7.8 to 11.0)	1.31 (1.26–1.36)	<0.0001
Persistent cough (R05)	44.1%	26.8%	17.3% (15.6 to 19.0)	2.14 (2.04–2.25)	<0.0001
Arrhythmia/abnormal heartbeat (R00, I49)	34.8%	24.2%	10.6% (8.9 to 12.3)	1.46 (1.39–1.53)	<0.0001

* Adjusted HRs from Cox proportional-hazards models adjusted for any residual post-matching imbalance.

**Table 4:** Lifetime cumulative incidence and Adjusted Hazard Ratios for clinical outcomes in propensity-score-matched survivors of leukemia (1:1 Matching, *n* = 5016). (Adjusted Hazard Ratio (Adj HR)) ICD codes provided for selected variables. (Any respiratory disease, Influenza and pneumonia, circulatory system disease).

Outcome	Mucositis (*n* = 2508)	No Mucositis (*n* = 2508)	Absolute Difference, % (95% CI)	Adjusted HR (95% CI) *	*p*-Value
All-cause mortality	52.3%	48.6%	3.7% (1.4 to 6.0)	1.12 (1.05–1.19)	<0.0001
Dysphagia (R13.1×)	9.8%	8.3%	1.5% (−0.1 to 3.1)	1.18 (1.07–1.30)	0.001
Malnutrition (E40–E46)	18.4%	15.1%	3.3% (1.6 to 5.0)	1.24 (1.12–1.37)	<0.0001
Any respiratory system disease (J00–J99)	71.2%	69.4%	1.8% (−0.3 to 3.9)	1.09 (1.03–1.15)	0.002
Influenza and pneumonia (J09–J18)	48.1%	46.7%	1.4% (−0.9 to 3.7)	1.06 (1.00–1.13)	0.06
Circulatory system disease (I00–I99)	62.5%	61.8%	0.7% (−1.8 to 3.2)	1.03 (0.97–1.09)	0.32
Persistent cough (R05)	28.4%	27.1%	1.3% (−0.8 to 3.4)	1.11 (1.04–1.18)	0.002
Arrhythmia/abnormal heartbeat (R00, I49)	31.2%	29.8%	1.4% (−0.9 to 3.7)	1.05 (0.98–1.12)	0.18

* Adjusted HRs from Cox proportional-hazards models adjusted for any residual post-matching imbalance.

**Table 5:** Formal test of effect modification by cancer type: ratio of Adjusted Hazard Ratios and *p* for interaction.

Outcome	CLOP HR (95% CI)	Leukemia HR (95% CI)	Ratio of HRs (95% CI)	*p* Interaction
All-cause mortality	1.94 (1.87–2.01)	1.12 (1.05–1.19)	1.73 (1.61–1.86)	<0.0001
Dysphagia	3.42 (3.28–3.57)	1.18 (1.07–1.30)	2.90 (2.62–3.21)	<0.0001
Malnutrition	2.81 (2.66–2.97)	1.24 (1.12–1.37)	2.27 (2.04–2.52)	<0.0001
Any respiratory disease	1.68 (1.63–1.73)	1.09 (1.03–1.15)	1.54 (1.45–1.54)	<0.0001
Influenza/pneumonia	1.79 (1.72–1.86)	1.79 (1.72–1.86)	1.69 (1.61–1.77)	<0.0001
Circulatory disease	1.31 (1.26–1.31)	1.03 (0.97–1.03)	1.27 (1.20–1.34)	<0.0001

Adjusted HRs from Cox proportional-hazards models adjusted for any residual post-matching imbalance. All interaction values are <0.0001, confirming highly significant effect modification by cancer type. (Adjusted Hazard Ratio (Adj HR)).

## Data Availability

This retrospective comparative cohort study utilized the TriNetX Global Health Research Network (data accessed 10 December 2025), a federated, HIPAA-compliant database that aggregates de-identified longitudinal electronic health records from over 90 predominantly U.S. healthcare organizations, encompassing more than 110 million patients.

## Supplementary Materials

The following supporting information can be downloaded at: https://www.mdpi.com/article/10.3390/medsci14010142/s1. Table S1. Sensitivity Analysis 1: Restricted to Index Events After 1 January 2015 (IMRT Era), Table S2. Sensitivity Analysis 2: Mucositis Diagnosis Required Within 2 Years of Cancer History Code, Table S3. Sensitivity Analysis 3: Tighter Caliper Matching (0.05 instead of 0.1), Table S4. Sensitivity Analysis 4: Exclusion of Mucositis Diagnosed >5 Years After Cancer History Code, Table S5. Timing of First Severe Oral Mucositis Diagnosis Relative to Index Date (Cancer History Code) in the Primary Matched Cohorts, Figure S1. Propensity Score Density Plot—Cancer of the Lip, Oral Cavity, and Pharynx Cohort, Figure S2. Propensity Score Density Plot—Leukemia Cohort.

## References

[1] Ang K.K., Harris J., Wheeler R., Weber R., Rosenthal D.I., Nguyen-Tân P.F., Westra W.H., Chung C.H., Jordan R.C., Lu C. (2010). Human papillomavirus and survival of patients with oropharyngeal cancer. N. Engl. J. Med..

[2] Gillison M.L., Trotti A.M., Harris J., Eisbruch A., Harari P.M., Adelstein D.J., Jordan R.C.K., Zhao W., Sturgis E.M., Burtness B. (2019). Radiotherapy plus cetuximab or cisplatin in human papillomavirus-positive oropharyngeal cancer (NRG Oncology RTOG 1016): A randomised, multicentre, non-inferiority trial. Lancet.

[3] Dietz A., Taylor K., Bayer O., Singer S., Follmann M., Nothacker M., Langer T., Klussmann P., Lang S., Hoffmann T. (2025). Evidence-based guideline diagnosis, treatment, prevention and aftercare of oropharyngeal and hypopharyngeal carcinoma. Ger. Med. Sci..

[4] Eisbruch A., Ten Haken R.K., Kim H.M., Marsh L.H., Ship J.A. (1999). Dose, volume, and function relationships in parotid salivary glands following conformal and intensity-modulated irradiation of head and neck cancer. Int. J. Radiat. Oncol. Biol. Phys..

[5] Short N.J., Zhou S., Fu C., Berry D.A., Walter R.B., Freeman S.D., Hourigan C.S., Huang X., Nogueras Gonzalez G., Hwang H. (2020). Association of Measurable Residual Disease with Survival Outcomes in Patients with Acute Myeloid Leukemia: A Systematic Review and Meta-analysis. JAMA Oncol..

[6] Djuric M., Hillier-Kolarov V., Belic A., Jankovic L. (2006). Mucositis prevention by improved dental care in acute leukemia patients. Support Care Cancer.

[7] Anderson C., Saunders D. (2025). Oral Mucositis in Head and Neck Cancer Patients. Semin. Radiat. Oncol..

[8] Wygoda A., Rutkowski T., Hutnik M., Składowski K., Goleń M., Pilecki B. (2013). Acute mucosal reactions in patients with head and neck cancer. Three patterns of mucositis observed during radiotherapy. Strahlenther. Onkol..

[9] Martinez J.M., Pereira D., Chacim S., Mesquita E., Sousa I., Martins Â., Azevedo T., Mariz J.M. (2014). Mucositis care in acute leukemia and non-Hodgkin lymphoma patients undergoing high-dose chemotherapy. Support Care Cancer.

[10] Abdalla-Aslan R., Bonomo P., Keefe D., Blijlevens N., Cao K., Cheung Y.T., Fregnani E.R., Miller R., Raber-Durlacher J., MASCC Mucositis Study Group (2024). Guidance on mucositis assessment from the MASCC Mucositis Study Group and ISOO: An international Delphi study. eClinicalMedicine.

[11] Elad S., Cheng K.K.F., Lalla R.V., Yarom N., Hong C., Logan R.M., Bowen J., Gibson R., Saunders D.P., Mucositis Guidelines Leadership Group of the Multinational Association of Supportive Care in Cancer and International Society of Oral Oncology (MASCC/ISOO) (2020). MASCC/ISOO clinical practice guidelines for the management of mucositis secondary to cancer therapy. Cancer.

[12] Lalla R.V. (2020). Evidence-Based Management of Oral Mucositis. JCO Oncol. Pract..

[13] Bensinger W., Schubert M., Ang K.K., Brizel D., Brown E., Eilers J.G., Elting L., Mittal B.B., Schattner M.A., Spielberger R. (2008). NCCN Task Force Report: Prevention and management of mucositis in cancer care. J. Natl. Compr. Cancer Netw..

[14] Peterson D.E., Boers-Doets C.B., Bensadoun R.J., Herrstedt J., ESMO Guidelines Committee (2015). Management of oral and gastrointestinal mucosal injury: ESMO Clinical Practice Guidelines for diagnosis, treatment, and follow-up. Ann. Oncol..

[15] Bergamaschi L., Vincini M.G., Zaffaroni M., Pepa M., Angelicone I., Astone A., Bergamini C., Buonopane S., Conte M., De Rosa N. (2023). Management of radiation-induced oral mucositis in head and neck cancer patients: A real-life survey among 25 Italian radiation oncology centers. Support Care Cancer.

[16] Bossi P., Numico G., De Santis V., Redda M.G.R., Reali A., Belgioia L., Rocca M.C., Orlandi E., Airoldi M., Bacigalupo A. (2014). Prevention and treatment of oral mucositis in patients with head and neck cancer treated with (chemo) radiation: Report of an Italian survey. Support Care Cancer.

[17] Soutome S., Yanamoto S., Funahara M., Hasegawa T., Komori T., Yamada S.-I., Kurita H., Yamauchi C., Shibuya Y., Kojima Y. (2017). Effect of perioperative oral care on prevention of postoperative pneumonia associated with esophageal cancer surgery: A multicenter case-control study with propensity score matching analysis. Medicine.

[18] Crowder S.L., Douglas K.G., Yanina Pepino M., Sarma K.P., Arthur A.E. (2018). Nutrition impact symptoms and associated outcomes in post-chemoradiotherapy head and neck cancer survivors: A systematic review. J. Cancer Surviv..

[19] Platek M.E., Reid M.E., Wilding G.E., Jaggernauth W., Rigual N.R., Hicks W.L., Popat S.R., Warren G.W., Sullivan M., Thorstad W.L. (2011). Pretreatment nutritional status and locoregional failure of patients with head and neck cancer undergoing definitive concurrent chemoradiation therapy. Head Neck.

[20] De Neve N.Y., Benoit D.D., Depuydt P.O., Offner F.C., Nollet J., Noens L.A., Decruyenaere J.M. (2010). Aspiration pneumonia: An underestimated cause of severe respiratory failure in patients with haematological malignancies and severe oral mucositis?. Acta Clin. Belg..

[21] Vainshtein J.M., Moon D.H., Feng F.Y., Chepeha D.B., Eisbruch A., Stenmark M.H. (2015). Long-term quality of life after swallowing and salivary-sparing chemo-intensity modulated radiation therapy in survivors of human papillomavirus-related oropharyngeal cancer. Int. J. Radiat. Oncol. Biol. Phys..

[22] Xu B., Boero I.J., Hwang L., Le Q.T., Moiseenko V., Sanghvi P.R., Cohen E.E.W., Mell L.K., Murphy J.D. (2015). Aspiration pneumonia after concurrent chemoradiotherapy for head and neck cancer. Cancer.

[23] Buchberger A.M.S., Strzelczyk E.A., Wollenberg B., Combs S.E., Pickhard A., Pigorsch S.U. (2021). Report on Late Toxicity in Head-and-Neck Tumor Patients with Long Term Survival after Radiochemotherapy. Cancers.

[24] Hutcheson K.A., Lewin J.S., Barringer D.A., Lisec A., Gunn G.B., Moore M.W., Holsinger F.C. (2012). Late dysphagia after radiotherapy-based treatment of head and neck cancer. Cancer.

[25] Feng F.Y., Kim H.M., Lyden T.H., Haxer M.J., Worden F.P., Feng M., Moyer J.S., Prince M.E., Carey T.E., Wolf G.T. (2010). Intensity-modulated chemoradiotherapy aiming to reduce dysphagia in patients with oropharyngeal cancer: Clinical and functional results. J. Clin. Oncol..

[26] Christianen M.E., Schilstra C., Beetz I., Muijs C.T., Chouvalova O., Burlage F.R., Doornaert P., Koken P.W., Leemans C.R., Rinkel R.N. (2012). Predictive modelling for swallowing dysfunction after primary (chemo)radiation: Results of a prospective observational study. Radiother. Oncol..

[27] Starmer H., Gourin C., Lua L.L., Burkhead L. (2011). Pretreatment swallowing assessment in head and neck cancer patients. Laryngoscope.

[28] Hutcheson K.A., Yuk M., Hubbard R., Gunn G.B., Fuller C.D., Lai S.Y., Lin H., Garden A.S., Rosenthal D.I., Hanna E.Y. (2017). Delayed lower cranial neuropathy after oropharyngeal intensity-modulated radiotherapy: A cohort analysis and literature review. Head Neck.

[29] Dirix P., Abbeel S., Vanstraelen B., Hermans R., Nuyts S. (2009). Dysphagia after chemoradiotherapy for head-and-neck squamous cell carcinoma: Dose-effect relationships for the swallowing structures. Int. J. Radiat. Oncol. Biol. Phys..

[30] Beynon R.A., Lang S., Schimansky S., Penfold C.M., Waylen A., Thomas S.J., Pawlita M., Waterboer T.W., Martin R.M., May M. (2018). Tobacco smoking and alcohol drinking at diagnosis of head and neck cancer and all-cause mortality: Results from head and neck 5000, a prospective observational cohort of people with head and neck cancer. Int. J. Cancer.

[31] Forastiere A.A., Zhang Q., Weber R.S., Maor M.H., Goepfert H., Pajak T.F., Morrison W., Glisson B., Trotti A., Ridge J.A. (2013). Long-term results of RTOG 91-11: A comparison of three nonsurgical treatment strategies to preserve the larynx in patients with locally advanced larynx cancer. J. Clin. Oncol..

[32] Mell L.K., Torres-Saavedra P.A., Wong S.J., A Kish J., Chang S.S., Jordan R.C., Liu T., Truong M.T., Winquist E.W., Takiar V. (2024). Radiotherapy with cetuximab or durvalumab for locoregionally advanced head and neck cancer in patients with a contraindication to cisplatin (NRG-HN004): An open-label, multicentre, parallel-group, randomised, phase 2/3 trial. Lancet Oncol..

[33] Mehanna H., Robinson M., Hartley A., Kong A., Foran B., Fulton-Lieuw T., Dalby M., Mistry P., Sen M., De-ESCALaTE HPV Trial Group (2019). Radiotherapy plus cisplatin or cetuximab in low-risk human papillomavirus-positive oropharyngeal cancer (De-ESCALaTE HPV): An open-label randomised controlled phase 3 trial. Lancet.

[34] Szczesniak M.M., Maclean J., Zhang T., Graham P.H., Cook I.J. (2014). Persistent dysphagia after head and neck radiotherapy: A common and under-reported complication with significant effect on non-cancer-related mortality. Clin. Oncol..

[35] Baudelet M., Van den Steen L., Tomassen P., Bonte K., Deron P., Huvenne W., Rottey S., De Neve W., Sundahl N., Van Nuffelen G. (2019). Very late xerostomia, dysphagia, and neck fibrosis after head and neck radiotherapy. Head Neck.

[36] Meng N.H., Li C.I., Hua C.H., Lin T.C., Chiu C.J., Lin C.L., Tsai M.H., Chiu P.J., Chang W.D., Tsou Y.A. (2023). Longitudinal changes in swallowing function after surgery and proactive swallowing therapy for oral cancer. Head Neck.

[37] Strojan P., Hutcheson K.A., Eisbruch A., Beitler J.J., Langendijk J.A., Lee A.W.M., Corry J., Mendenhall W.M., Smee R., Rinaldo A. (2017). Treatment of late sequelae after radiotherapy for head and neck cancer. Cancer Treat. Rev..

[38] Wishart L.R., Harris G.B., Cassim N., Alimin S., Liao T., Brown B., Ward E.C., Nund R.L. (2022). Association Between Objective Ratings of Swallowing and Dysphagia-Specific Quality of Life in Patients Receiving (Chemo)radiotherapy for Oropharyngeal Cancer. Dysphagia.

[39] Rubenstein E.B., Peterson D.E., Schubert M., Keefe D., McGuire D., Epstein J., Elting L.S., Fox P.C., Mucositis Study Section of the Multinational Association for Supportive Care in Cancer, International Society for Oral Oncology (2004). Clinical practice guidelines for the prevention and treatment of cancer therapy-induced oral and gastrointestinal mucositis. Cancer.

[40] Iglesias-Bartolome R., Uchiyama A., Molinolo A.A., Abusleme L., Brooks S.R., Callejas-Valera J.L., Edwards D., Doci C., Asselin-Labat M.-L., Onaitis M.W. (2018). Transcriptional signature primes human oral mucosa for rapid wound healing. Sci. Transl. Med..

[41] Maria O.M., Eliopoulos N., Muanza T. (2017). Radiation-Induced Oral Mucositis. Front. Oncol..

[42] Fakhry C., Zhang Q., Gillison M.L., Nguyen-Tân P.F., Rosenthal D.I., Weber R.S., Lambert L., Trotti A.M., Barrett W.L., Thorstad W.L. (2019). Validation of NRG oncology/RTOG-0129 risk groups for HPV-positive and HPV-negative oropharyngeal squamous cell cancer: Implications for risk-based therapeutic intensity trials. Cancer.

[43] Kawai S., Yokota T., Onozawa Y., Hamauchi S., Fukutomi A., Ogawa H., Onoe T., Onitsuka T., Yurikusa T., Todaka A. (2017). Risk factors for aspiration pneumonia after definitive chemoradiotherapy or bio-radiotherapy for locally advanced head and neck cancer: A monocentric case control study. BMC Cancer.

[44] Silverman D.A., Lin C., Tamaki A., Puram S.V., Carrau R.L., Seim N.B., Eskander A., Rocco J.W., Old M.O., Kang S.Y. (2020). Respiratory and pulmonary complications in head and neck cancer patients: Evidence-based review for the COVID-19 era. Head Neck.

[45] Patil V., Noronha V., Shrirangwar S., Menon N., Abraham G., Chandrasekharan A., Prabhash K. (2021). Aspiration pneumonia in head and neck cancer patients undergoing concurrent chemoradiation from India: Findings from a post hoc analysis of a phase 3 study. Cancer Med..

[46] Lu L., Li F., Gao Y., Kang S., Li J., Guo J. (2024). Microbiome in radiotherapy: An emerging approach to enhance treatment efficacy and reduce tissue injury. Mol. Med..

[47] Ciernikova S., Sevcikova A., Mladosievicova B., Mego M. (2023). Microbiome in Cancer Development and Treatment. Microorganisms.

[48] Wong J.L., Evans S.E. (2017). Bacterial Pneumonia in Patients with Cancer: Novel Risk Factors and Management. Clin. Chest Med..

[49] van den Berg M.G., Rütten H., Rasmussen-Conrad E.L., Knuijt S., Takes R.P., van Herpen C.M., Wanten G.J., Kaanders J.H., Merkx M.A. (2014). Nutritional status, food intake, and dysphagia in long-term survivors with head and neck cancer treated with chemoradiotherapy: A cross-sectional study. Head Neck.

[50] Mulasi U., Vock D.M., Jager-Wittenaar H., Teigen L., Kuchnia A.J., Jha G., Fujioka N., Rudrapatna V., Patel M.R., Earthman C.P. (2020). Nutrition Status and Health-Related Quality of Life Among Outpatients with Advanced Head and Neck Cancer. Nutr. Clin. Pract..

[51] Vangelov B., Venchiarutti R.L., Smee R.I. (2017). Critical Weight Loss in Patients with Oropharynx Cancer During Radiotherapy (±Chemotherapy). Nutr. Cancer.

[52] Colevas A.D., Cmelak A.J., Pfister D.G., Spencer S., Adkins D., Birkeland A.C., Brizel D.M., Busse P.M., Caudell J.J., Durm G. (2025). NCCN Guidelines^®^ Insights: Head and Neck Cancers, Version 2.2025. J. Natl. Compr. Cancer Netw..

[53] Zadik Y., Arany P.R., Fregnani E.R., Bossi P., Antunes H.S., Bensadoun R.-J., Gueiros L.A., Majorana A., Nair R.G., Mucositis Study Group of the Multinational Association of Supportive Care in Cancer/International Society of Oral Oncology (MASCC/ISOO) (2019). Systematic review of photobiomodulation for the management of oral mucositis in cancer patients and clinical practice guidelines. Support. Care Cancer.

[54] Antunes H.S., Herchenhorn D., Small I.A., Araújo C.M., Viégas C.M.P., Cabral E., Rampini M.P., Rodrigues P.C., Silva T.G., Ferreira E.M. (2013). Phase III trial of low-level laser therapy to prevent oral mucositis in head and neck cancer patients treated with concurrent chemoradiation. Radiother. Oncol..

[55] Evangelidis P., Salvaras G., Vardi A., Leonidis I., Papakonstantinou A., Sakellari I., Gavriilaki E. (2026). Treosulfan-based conditioning strategies in older or frail patients undergoing allogeneic hematopoietic cell transplantation. Expert Rev. Hematol..

