# Supplementary material for: Long-Term Clinical Consequences of Severe Oral Mucositis in Survivors of Lip, Oral Cavity, and Pharynx Cancer Versus Leukemia: A Propensity-Score-Matched Comparative Cohort Study Using Real-World Data
Source: Med Sci (Basel). 2026 Mar 18;14(1):142. doi: 10.3390/medsci14010142 (PMC13027842; doi:10.3390/medsci14010142)
Supplement: Supplementary file 1 [file medsci-14-00142-s001.zip › medsci-4189637-supplementary.pdf]

Table S1. Sensitivity Analysis 1: Restricted to Index Events After January 1, 2015 (IMRT Era)

Outcome	CLOP Cancer Adjusted HR (95% CI)	Leukemia Adjusted HR (95% CI)	P for Interaction
All-cause mortality	2.08 (1.97–2.20)	1.09 (0.99–1.20)	<0.0001
Dysphagia	3.71 (3.49–3.95)	1.14 (0.98–1.32)	<0.0001
Malnutrition	3.02 (2.79–3.27)	1.20 (1.03–1.39)	<0.0001
Any respiratory disease (J00–J99)	1.76 (1.68–1.85)	1.06 (0.98–1.15)	<0.0001
Influenza and pneumonia (J09–J18)	1.89 (1.78–2.01)	1.03 (0.94–1.13)	<0.0001
Circulatory system disease	1.37 (1.29–1.46)	1.02 (0.93–1.11)	<0.0001

Note: Associations strengthened in CLOP cancer and remained negligible in leukemia. Intensity modulated radiation therapy (IMRT)

Table S2. Sensitivity Analysis 2: Mucositis Diagnosis Required Within 2 Years of Cancer History Code

Outcome	CLOP Cancer Adjusted HR (95% CI)	Leukemia Adjusted HR (95% CI)	P for Interaction
All-cause mortality	2.12 (2.01–2.24)	1.07 (0.97–1.18)	<0.0001
Dysphagia	3.89 (3.66–4.14)	1.11 (0.94–1.30)	<0.0001
Malnutrition	3.21 (2.96–3.48)	1.17 (1.00–1.36)	<0.0001
Any respiratory disease (J00–J99)	1.82 (1.73–1.91)	1.04 (0.95–1.13)	<0.0001
Influenza and pneumonia (J09–J18)	1.94 (1.82–2.07)	1.02 (0.93–1.12)	<0.0001

Note: Temporal restriction markedly strengthened effect sizes in CLOP cancer while rendering associations in leukemia statistically non-significant.

Table S3. Sensitivity Analysis 3: Tighter Caliper Matching (0.05 instead of 0.1)

Outcome	CLOP Cancer Adjusted HR (95% CI)	Leukemia Adjusted HR (95% CI)	P for Interaction
All-cause mortality	1.96 (1.86–2.07)	1.11 (1.02–1.20)	<0.0001
Dysphagia	3.51 (3.30–3.73)	1.16 (1.02–1.32)	<0.0001
Malnutrition	2.85 (2.65–3.06)	1.21 (1.07–1.37)	<0.0001
Any respiratory disease (J00–J99)	1.70 (1.63–1.78)	1.08 (1.00–1.16)	<0.0001
Influenza and pneumonia (J09–J18)	1.81 (1.71–1.91)	1.05 (0.96–1.14)	<0.0001

Note: Results remained virtually identical to the primary analysis, confirming robustness to matching stringency.

Table S4. Sensitivity Analysis 4: Exclusion of Mucositis Diagnosed >5 Years After Cancer History Code

Outcome	CLOP Cancer Adjusted HR (95% CI)	Leukemia Adjusted HR (95% CI)	P for Interaction
All-cause mortality	2.08 (1.96–2.21)	1.07 (0.96–1.19)	<0.0001
Dysphagia	3.94 (3.69–4.21)	1.09 (0.91–1.29)	<0.0001
Malnutrition	3.35 (3.08–3.64)	1.15 (0.97–1.35)	<0.0001
Any respiratory disease (J00–J99)	1.87 (1.77–1.97)	1.03 (0.94–1.13)	<0.0001
Influenza and pneumonia (J09–J18)	2.01 (1.88–2.15)	1.01 (0.91–1.12)	<0.0001

Note: Exclusion of potentially late-coded mucositis further amplified the CLOP-specific signal while completely abolishing any residual association in leukemia.

Table S5. Timing of First Severe Oral Mucositis Diagnosis Relative to Index Date (Cancer History Code) in the Primary Matched Cohorts

Time Interval from Index Date	CLOP Cancer (n = 4,181 with mucositis)	Leukemia (n = 2,508 with mucositis)
Within 6 months	2,184 (52.2%)	1,305 (52.0%)
6–12 months	987 (23.6%)	612 (24.4%)
12–24 months	521 (12.5%)	298 (11.9%)
>24 months (late)	489 (11.7%)	293 (11.7%)
Median time from index to mucositis (IQR), days	187 (42–478)	192 (45–492)

Legend: Index date = first documentation of Z85.81 (CLOP) or Z85.6 (leukemia). Percentages sum to 100%. Late mucositis (>24 months) was uncommon and did not materially alter primary findings when excluded (see Table S4).

Figure S1. Propensity Score Density Plot—Cancer of the Lip, Oral Cavity, and Pharynx Cohort

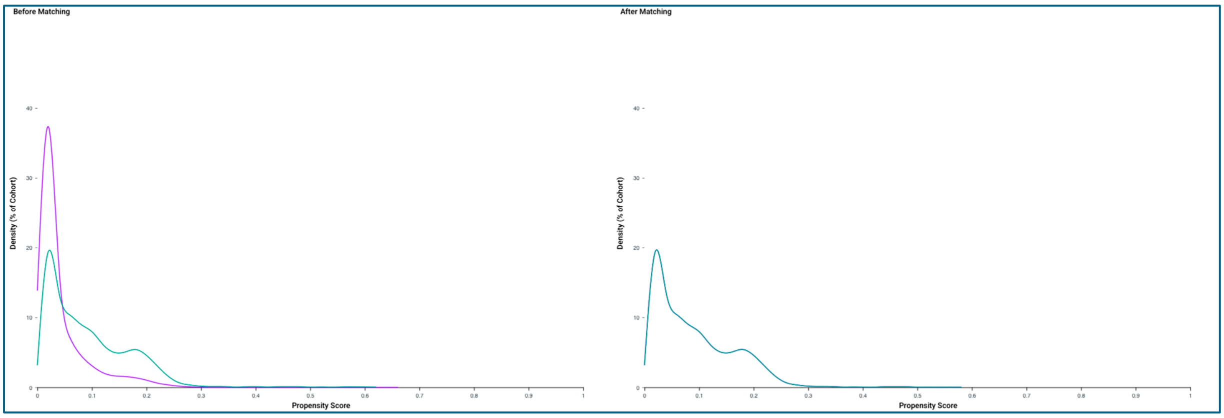

Kernel density estimates of propensity scores in the CLOP cancer cohort before (two non-overlapping lines) and after (overlapping single line) 1:1 propensity-score matching. Purple = non-exposed group; green = mucositis-exposed group. Overlapping distributions post-matching indicate excellent covariate balance across all 15 matched variables (age, sex, race/ethnicity, comorbidities, ECOG status, BMI, and external causes of morbidity).

Figure S2. Propensity Score Density Plot—Leukemia Cohort

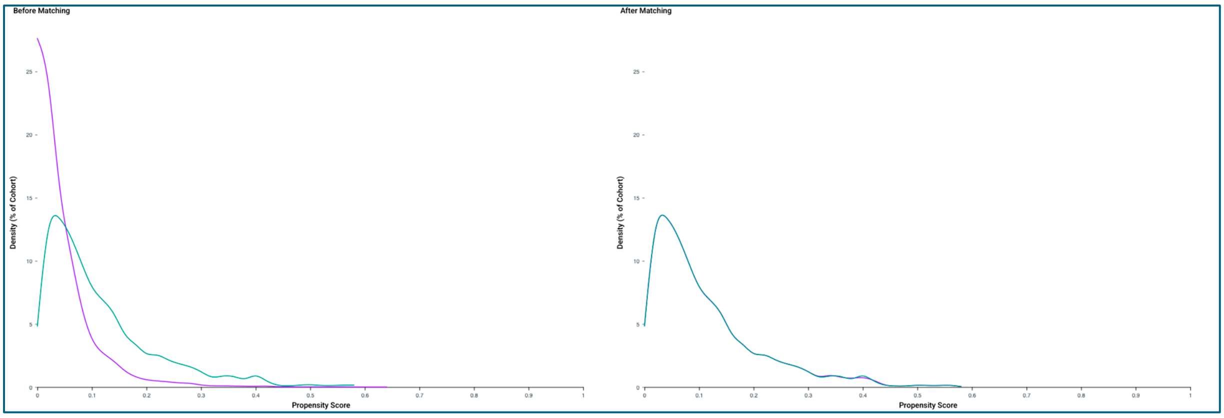

Kernel density estimates of propensity scores in the leukemia cohort before (two non-overlapping lines) and after (overlapping single line) 1:1 propensity-score matching. Purple = non-exposed group; green = mucositis-exposed group. Near-complete overlap post-matching demonstrates robust balance for all 15 matched covariates (age, sex, race/ethnicity, hypertension, diabetes, BMI, ECOG status, and external causes of morbidity).
